# Structure-Based Modeling of the Gut Bacteria–Host Interactome Through Statistical Analysis of Domain–Domain Associations Using Machine Learning

**DOI:** 10.3390/biotech14010013

**Published:** 2025-02-25

**Authors:** Despoina P. Kiouri, Georgios C. Batsis, Thomas Mavromoustakos, Alessandro Giuliani, Christos T. Chasapis

**Affiliations:** 1 Institute of Chemical Biology, National Hellenic Research Foundation, 11635 Athens, Greece; despoina.kiouri.99@gmail.com (D.P.K.); georgebatsis95@gmail.com (G.C.B.); 2 Laboratory of Organic Chemistry, Department of Chemistry, National and Kapodistrian University of Athens, 15772 Athens, Greece; tmavrom@chem.uoa.gr; 3 Environment and Health Department, Istituto Superiore di Sanità, 00161 Rome, Italy; alessandro.giuliani@iss.it

**Keywords:** gut microbiome, protein networks, domain interactions, host–bacteria interactions, machine learning

## Abstract

The gut microbiome, a complex ecosystem of microorganisms, plays a pivotal role in human health and disease. The gut microbiome’s influence extends beyond the digestive system to various organs, and its imbalance is linked to a wide range of diseases, including cancer and neurodevelopmental, inflammatory, metabolic, cardiovascular, autoimmune, and psychiatric diseases. Despite its significance, the interactions between gut bacteria and human proteins remain understudied, with less than 20,000 experimentally validated protein interactions between the host and any bacteria species. This study addresses this knowledge gap by predicting a protein–protein interaction network between gut bacterial and human proteins. Using statistical associations between Pfam domains, a comprehensive dataset of over one million experimentally validated pan-bacterial–human protein interactions, as well as inter- and intra-species protein interactions from various organisms, were used for the development of a machine learning-based prediction method to uncover key regulatory molecules in this dynamic system. This study’s findings contribute to the understanding of the intricate gut microbiome–host relationship and pave the way for future experimental validation and therapeutic strategies targeting the gut microbiome interplay.

## 1. Introduction

Recently, the entire gut microecosystem has been considered as an essential organ and major regulator of the human body. It is estimated that the gut microbiome (GM) is comprised of more than 1014 microorganisms, according to the publicly available genomic and proteomic microbiome databases [1]. These bacteria, viruses, fungi, archaea, and protists coexist and interact in a complex system [1].

To further emphasize the size and complexity of the GM, it is estimated that 3 million genes in the GM encode enzymes that produce thousands of metabolites, while the human genome only contains about 23,000 genes [2]. Most of the bacteria that are part of the GM form symbiotic relationships with the host and are essential for the establishment and balance of both the intestinal innate and adaptive immunity and, by extension, the maintenance of host homeostasis [3]. In the bacterial population of the GM, only a few phyla are present, with the most prevalent being *Firmicutes* and *Bacteroidetes*, which represent 90% of the bacterial colonies of the GM [4], and *Actinobacteria*, *Proteobacteria*, *Fusobacteria*, and *Verrucomicrobia* representing the remaining 10% [5].

Being key players, the bacterial populations of the GM not only affect the entire digestive tract, but also various lateral organs, including the liver, brain, and pancreas [6]. For that reason, bacterial gut microbiota deviations have been associated with a broad range of intestinal and extraintestinal disorders, including neurodevelopmental [7], inflammatory [8], metabolic [9], cardiovascular [10], autoimmune [11], and psychiatric diseases [12], as well as cancer [13]. Consequently, any alterations in the symbiotic relationship between the intestinal flora and the host can promote the development of gut-related pathological conditions [14].

The most common gastrointestinal (GI) disorders that are influenced by the GM composition are intestinal bowel diseases (IBDs), irritable bowel syndrome (IBS), coeliac disease, and chronic liver and pancreatic disorders [15]. The bacteria of the GM have also been linked with various metabolic syndromes, like insulin resistance, high blood pressure, and cardiovascular diseases [16]. They also play a key role in neural development via a sophisticated bidirectional communication, commonly referred to as the gut–brain axis [16]. Gut dysbiosis has been linked with several neuropsychiatric disorders (NPDs), including autism spectrum disorder (ASD), attention-deficit/hyperactivity disorder (ADHD), and schizophrenia (SCZ) [16]. Additionally, the GM composition is believed to contribute to the development of neuropsychiatric disorders, like depression, Parkinson’s, Alzheimer’s, and Huntington’s disease [17,18]. Moreover, several rather complex autoimmune disorders, such as rheumatoid arthritis (RA), spondyloarthritis (SpA), systemic lupus erythematosus (SLE), multiple sclerosis (MS), and type 1 diabetes (T1D) are influenced by dysbiotic microbiota in the gut environment [11,19]. Furthermore, the GM is involved in the formation and progression of different types of cancer, including esophageal, liver, pancreatic, and, most significantly, colorectal cancer (CRC), as well as their response to various systemic therapies, because these bacteria and their secretary metabolites may also interfere with the pharmacodynamics of anti-cancer medications [6,20].

Recent studies have focused on the experimental determination of human and bacterial intra-species protein interactions. The physical interactions between the proteins of *Bacillus anthracis*, *Francisella tularensis*, *Yersinia pestis*, *Mycobacterium tuberculosis*, and the host’s proteins have been identified by high-throughput yeast two-hybrid assays [21,22,23]. Li et al. have also used photo-ANA (i.e., a bifunctional amino acid with a bio-orthogonal handle and a photoreactive warhead) for the recognition of protein interactions between *Salmonella enterica serovar Typhimurium* and the human host [24]. In order to investigate protein interactions in a more native environment than yeast-two-hybrid methods, assays based on mass spectrometry (MS) and cross-linking were developed lately to identify host–bacteria interactions between human and *Salmonella enterica*, *Acinetobacter baumanii*, and *Lactobacillus acidophilus* proteins [25,26,27]. Given the numerous obstacles associated with experimental approaches for deciphering the mysteries of the human gut microbiota, computational methodologies have been developed as a first step in addressing the perplexity of this inter-species dynamic system. Computational approaches, from domain–domain interaction predictions to sophisticated machine learning (ML) algorithms, have further expanded our capability to predict protein–protein interactions (PPIs), offering insights into the structural and functional dynamics of the GM [28,29,30,31,32].

Given the current state of knowledge, there is a scarcity of experimental studies that have successfully identified interactions between proteins from the bacteria of the GM and the human host, despite the presence of public databases containing experimental data on interactions between bacterial species and humans. This research gap may impede our understanding of how imbalances in the relationship between GM bacteria and humans contribute to the development of diseases. To get a better idea of the experimental data availability, the experimentally validated pan human–bacterial protein interaction network was calculated from data that were retrieved from public databases (i.e., HPIDB [33,34], IntAct [35], PHISTO [36], and MorCVD [37]). To this day, this network contains less than 20,000 interactions. Nevertheless, the entire GM is thought to be comprised of 300 to 500 different bacterial species, so it is safe to say that the interactions between them and the host proteins are really understudied due to lack of data. Recognizing the challenges inherent in experimental methods and the limited data on host–gut interactions, we sought to leverage the power of ML methodologies to predict these relationships from readily available domain information [38,39]. Thus, in this study, a protein–protein interaction network (PPIN) between gut bacterial and human proteins was predicted by utilizing ML to extract significant associations between Pfam domains [40], which were derived from a comprehensive publicly available dataset containing 1,100,787 experimentally validated pan-bacterial–human PPIs and human PPIs, as well as inter- and intra- species PPIs from different organisms including viruses, plants, and animals. This PPIN was subsequently analyzed to uncover the most influential regulatory molecules that are key players of this dynamic system [41].

## 2. Materials and Methods

### 2.1. Data Collection

#### 2.1.1. Protein Interaction Dataset

##### Experimental Protein Interaction Data

First, all publicly available experimental pan human–bacterial PPI data, which contain 19,686 interactions between 5714 bacterial and 4287 human proteins, were retrieved from four public databases: HPIDB [33,34], IntAct [35], PHISTO [36], and MorCVD [37] (original dataset). Another, more inclusive PPI dataset, which contained interactions from 6 widely used interaction databases (i.e., IntAct [35], MINT [42], DIP [43], HPRD [44], BioGRID [45], and SIFTS [46,47]) was also obtained. This inclusive extended PPI dataset contains 1,081,401 PPIs, out of which 330,530 are human inter-species PPIs and 750,871 are inter- and intra-species interactions of different organisms, including bacteria, viruses, plants, and animals. All the proteins of the original and larger dataset were also mapped to their Pfam IDs using the UniProt Application Programming Interface (API) [48].

##### Dataset Preprocessing for Machine Learning

For the construction of the dataset, both the original and the larger PPI datasets were then filtered and only the interactions where both participating proteins were matched to Pfam IDs were kept. To create a consistent dataset for the development of the proposed ML model, the negative (non-interaction) sampling was an essential step. Towards this, two additional data collections were utilized: the complete human proteome was retrieved from UniProt Proteomes, and the tissue topology of every individual protein was then obtained from the Human Protein Atlas [49]. A dataset (golden standard dataset) containing 17,278 experimentally supported domain–domain interactions from PDB complexes was retrieved from the 3did database [32,50]. The negative dataset comprised human proteins known to not interact. These proteins were selected based on two criteria: they are expressed exclusively in different human organs, and their domains (i.e., Pfam domains [51]) do not interact. Additionally, the potential non-PPIs were filtered and only those that do not exist in the positive dataset nor the available human interactome were kept.

##### Training, Validation, and Experimental Test Sets

For the development of ML pipelines, training, validation, and test datasets are indispensable for ensuring the model’s effective learning, optimization, and unbiased evaluation. The training dataset plays a pivotal role by enabling the model to learn patterns, relationships, and dependencies within the data. The validation dataset is critical for fine-tuning the model and optimizing hyperparameters, helping to mitigate overfitting or underfitting without influencing the final evaluation. The test dataset, reserved exclusively for the final assessment, provides an unbiased evaluation of the model’s performance on completely unseen data, serving as a measure of its real-world applicability and robustness. Specifically, performance metrics obtained from the test set reflect the model’s ability to accurately detect experimental interactions, which is essential for its intended application.

The positive and negative datasets were combined into a single large-scale dataset encompassing protein–protein interactions (PPIs) and non-interactions (non-PPIs). This combined dataset was systematically divided into three subsets: the training dataset, the validation dataset, and the test dataset. To ensure that the model could generalize effectively, the split was performed such that all possible domain–domain interactions (DDIs) between Pfam domains inferred from PPIs in the validation and test datasets were included in the training dataset. The dataset distribution is presented in Table 1. Since the primary goal of the model is to predict protein interactions between the host and gut bacteria, all experimentally supported PPIs between gut bacteria and human proteins localized in the gut (13 pairs) were isolated from the large-scale dataset and designated as an additional experimental test set. This set allows for a focused assessment of the model’s predictive capabilities in the specific context of host–gut bacteria interactions, thereby enhancing the evaluation’s relevance to its intended biological application.

#### 2.1.2. Gut Data Collection

The human proteome was then filtered using only the labels for gut and brain topologies from the Human Protein Atlas [49], and then only the entries with available Pfam IDs were kept (30,129 proteins). From the Human Gut Microbiome Atlas [52,53] all the bacterial strains that are labeled either as “Healthy” or “Unspecified” (since they have not been linked to any specific diseases) were chosen and were then mapped to their respected UniProt Proteome IDs. Subsequently, all the proteins that were included in each Proteome ID were retrieved using the Proteins API of the European Bioinformatics Institute (EBI) [54], and only the entries with available Pfam IDs were kept (553,872 proteins).

### 2.2. Machine Learning Model

**Figure 1:**
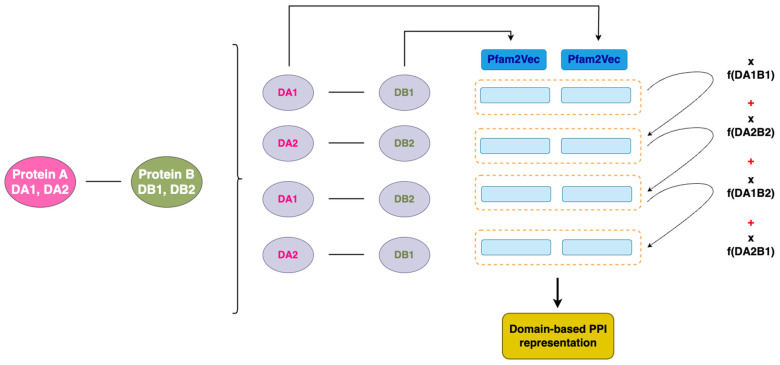
Pfam-based PPI representation from adapted Word2Vec.

An advanced ML method was implemented for the prediction of PPIs between the host and bacteria within the gut. The preprocessing step of the ML method development is the conversion of the data (in this case, Pfam IDs, which are in text format) to numeric representation since these models can only process numeric data. Word2Vec, a deep learning algorithm that extracts word embeddings (i.e., the numeric representation of a word), was employed to obtain vector representations of protein pairs [55]. In this study, a similar methodology to the one applied by Hannigan et al. [56] was used for the adaptation of Word2Vec to Pfam2Vec, where the words used for the embedding extraction are Pfam IDs. For the training of the Pfam2Vec model, a corpus (i.e., text dataset) was constructed from the training dataset, and the model’s hyperparameters used were retrieved from the work of Hannigan et al. [56]. A corpus is essentially a collection of texts made by sentences and words; so, in this case, each sentence of the corpus represents a protein pair, while each potential DDI is represented by two sequential tokens. Every protein pair is represented as the concatenation of the output of Pfam2Vec of all the possible domain pairs that could potentially describe the interaction (i.e., all the possible combinations of the Pfam domains of the two proteins). Essentially, for each predicted protein–protein interaction, we create a vector representation by combining the individual vectors of the domain pairs that might mediate the interaction. The contribution of each domain pair vector to the final representation is proportional to how often that domain pair is observed in interacting proteins in our training data (Figure 1).

Each PPI is represented as a 200-dimension vector (as proposed by Hannigan et al. [56]) and can be used as input to an ensemble ML algorithm for the classification of protein pairs as “Interacting” or “Non-Interacting” based on domain information.

Subsequently, a prediction model leveraging an ML classifier, specifically random forest (RF), was developed and trained with the dataset mentioned above. An RF classifier is a well-established algorithm which offers advantages in terms of accuracy and generalization capacity (i.e., it is really good at predicting unknown data), aligning with the complexities occurring in the proposed classification task [57]. Since hyperparameter tuning is a critical step in optimizing the RF model’s performance, parameter space was explored by employing a grid search and a validation dataset for optimal hyperparameter configuration. In other words, hyperparameter tuning is essential to find the best computational setup of the prediction model that is needed for ideal functionality. The parameters tuned in the case of the RF model are the number of trees and the maximum number of features for node splitting. The hyperparameter optimization results can be found in Supplementary Materials, File S1.

To test the RF model on completely unknown data, the training and validation datasets were concatenated, and the RF classifier was retrained using the best hyperparameters. After the model’s retraining process, using the chosen decision threshold (DT), the test dataset was used for PPI prediction. A classification model like RF gives as an output the probability of a sample belonging to a specific class, and thus the DT is a probability cut-off value for a sample to belong to a certain class with statistical significance.

### 2.3. Model Application—Host and Gut Bacteria PPIN

Upon completion of the training phase, this model was employed to predict the protein interactions between the human proteins of the gut and the proteins of the gut’s bacteria. For this purpose, two protein pools were established for both human and bacterial proteins, and each potential pair of proteins was used as input to the prediction model. To ascertain the biological relevance of these predictions, only those protein pairs exhibiting a high likelihood of interaction, approaching a probability of 1, were selected for inclusion in the PPI network.

### 2.4. PPIN Clustering and Vizualization

Since this network contains every human protein (including isoforms and recently discovered proteins with unknown function) and every bacterial protein from UniProt (including homologous proteins in various strains), this pan human–bacterial network was subsequently clustered to be analyzed. At first, the UniProt Reference Clusters database was used to filter redundant sequences. In this work, UniRef90, which clusters UniRef100 sequences (i.e., containing identical sequences and subfragments) using the many-against-many sequence searching (MMseqs2) algorithm [58] to create clusters of sequences that have at least 90% sequence identity and overlap with the longest sequence at least 80%, was used. To further cluster this network, the UniRef90 clusters were expanded to fuzzy clusters that contained proteins with 70% similar gene names and at least one identical Pfam ID, to group similar proteins that would essentially have similar functions. This final network was visualized using Cytoscape (version 3.10.1) [58].

### 2.5. Biological Network Analysis

After the networks’ construction, the degree (i.e., the number of edges connected to a node) and IVI, a new metric of biological influence of each node within a network, were then calculated for every node of every network (Equation (1)). IVI integrates the most significant network centrality measures (i.e., degree centrality, ClusterRank, LH index, neighborhood connectivity, betweenness centrality, and collective influence) in order to synergize their effects and simultaneously remove their biases to identify the most essential regulatory molecules in a network [59]. In other words, the nodes whose hubness and spreading scores are simultaneously high have the highest IVI scores and are the most influential of the network.

Equation (1). IVI

(1) $${IVI}_{i,\ell }=(\sum_{j\neq i}A_{ij}+H(k_{j_{1}},k_{j_{2}},...,k_{j_{k_{i}}})+\sum_{v\in N(i)}H(k_{y_{1}},k_{y_{2}},...,k_{y_{k_{v}}}))\times ((\frac{\sum_{v\in N(i)}N(v)}{N(i)})+\;f(c_{i})\sum_{j\in N(i)}\left(k_{j}^{out}+1\right))\times (\sum_{m\neq i,m\neq n,n\neq i}\frac{S_{mn}(i)}{S_{mn}}+(k_{i}-1)\sum_{j\in \delta B(i,\ell )}\left(k_{j}-1\right))),$$

where $A_{ij}$ is the adjacency matrix of the corresponding network, K, N and H are the degree/node, set of first-order neighbors/node and H index of node *i* respectively. $f(c_{i})$ is the local clustering effect of *i*, $S_{mn}$ are n of shortest paths between nodes (*m*,*n*), $S_{mn}(i)$ are n of shortest paths between nodes (*m*,*n*) through node *i* and δB(i,ℓ) is a set of nodes at distance ℓ from node *i*.

Additionally, the mean degree (i.e., the mean value of the degrees of all the proteins belonging to a bacterial strain) was calculated for each of the bacteria and the plot that associates the mean degree with the strain’s abundance was visualized. To get a better understanding of the network’s organization, we used a variety of metrics to measure its assortativity (i.e., the preference for a network’s nodes to attach to other similar ones) (Equations (2)–(4)), since biological networks, which were previously classed as disassortative, are revealed to be somewhat assortative with the use of other measures of assortativity than the assortativity coefficient, *r* (i.e., the Pearson correlation coefficient of degree between pairs of linked nodes). In this case, the degree of each node was chosen as a measure of similarity, and thus, apart from the assortativity coefficient, the degree distribution, *P*(*k*) (i.e., the fraction of nodes in the network with degree *k*) (Equation (5)), average degree of connectivity, $k_{nn,i}^{w}$ (i.e., the average nearest neighbor degree of nodes with degree *k*) (Equation (6)), and correlation profile, z-score (i.e., comparison of the joint probability *P*(*_K_*1, *_K_*2) of finding a link between two nodes with degree K1 and K2 with the corresponding probability *P_r_*(*_K_*1, *_K_*2) in randomized networks) (Equation (7)) were calculated and visualized [60,61]. The assortativity coefficient was also calculated for the clustered network after excluding the top 1% most connected nodes, to see whether the scores and plots change.

Equation (2). Assortativity coefficient.

(2) $$r=\frac{\sum_{jk}jk\left(e_{jk}-q_{j}q_{k}\right)}{\sigma_{q}^{2}}$$

Equation (3). $q_{k}$, distribution of remaining degree (i.e., all edges leaving the node, besides the edge that connects the pair).

(3) $$q_{k}=\frac{\left(k+1\right)p_{k+1}}{\sum_{j>1}jp_{j}}$$

Equation (4). $e_{jk}$, the joint probability distribution of the remaining degrees of the two vertices (in this case, since the graph is undirected it is symmetric).

(4) $$\sum_{jk}e_{jk}=1\;\mathrm{and}\;\sum_{j}e_{jk}=q_{k}$$

Equation (5). Degree distribution.

(5) $$P\left(k\right)=\frac{n_{k}}{n}$$

Equation (6). Average degree of connectivity.

(6) $$k_{nn,i}^{w}=\frac{1}{s_{i}}\sum_{j\in N\left(i\right)}w_{ij}k_{j},$$

$where\;s_{i}$ is the weighted degree of node i, $w_{ij}$ is the weight of the edge that links i and j, $N\left(i\right)$ are the neighbors of node i and w are the weights of the edges (in this case, w=1).

Equation (7). Correlation profile.

(7) $$Z=\frac{P\left(K_{1},K_{2}\right)-E\left[P_{r}\left(K_{1},K_{2}\right)\right]}{\sigma \left(K_{1},K_{2}\right)}$$

where $E\left[P_{r}\left(K_{1},K_{2}\right)\right]$ and $\sigma \left(K_{1},K_{2}\right)$ are the and mean and standard deviation of the degree distribution in 1000 experiments of randomized networks.

### 2.6. Biological Significance Analysis

The GO analysis of the human fuzzy clusters was retrieved as a means of functional protein annotation and molecular pathway participation discovery. Additionally, the percentage of human and bacterial nodes with intrinsic disorder was fetched from MobiDB and the visualized [62]. Finally, a plot that associates the degree of the human proteins in the available human interactome from the protein interaction knowledgebase (PICKLE) database [63], and the degree of the same human proteins in the host–gut network was also visualized. The Pearson correlation coefficient, which measures linear correlation between two variables, was calculated for the two plots.

## 3. Results

### 3.1. Model Evaluation Metrics

For the classification assessment of protein interactions within the dataset, a robust evaluation framework encompassing basic error metrics and composite metrics was established. The essential components of the performance evaluation are the number of correctly classified samples, Type I and Type II errors [64], i.e., true positives (*TP*), true negatives (*TN*), false positives (*FP*), and false negatives (*FN*). These metrics were derived from the comparison of the model’s predictions against the labels that corresponded to reality. Derived metrics such as accuracy (*ACC*) (Equation (8)), precision (*PREC*) (Equation (9)), recall (*REC*) (Equation (10)), and F1 Score (Equation (11)) are significant for a comprehensive assessment of the model’s performance:

Equation (8). Accuracy, the ratio of correctly classified instances to the total instances.

(8) $$\mathrm{ACC}=\frac{\mathrm{TP}+\mathrm{TN}}{\mathrm{Total}\;\mathrm{Samples}}$$

Equation (9). Precision, the ratio of correctly predicted positive observations to the total predicted positives.

(9) $$\mathrm{PREC}=\frac{TP}{TP+FP}$$

Equation (10). Recall, the ratio of correctly predicted positive observations to all observations in the actual class.

(10) $$\mathrm{REC}=\frac{TP}{TP+FN}$$

Equation (11). F1 Score, the harmonic mean of PREC and REC.

(11) $$F_{1}=2\cdot \frac{\mathrm{PREC}\times \mathrm{REC}}{\mathrm{PREC}+\mathrm{REC}}$$

The receiver operating characteristic (ROC) curve is a graphical representation of a model’s prediction capability by plotting the true positive rate (*TPR*) (Equation (12)) vs. the false positive rate (*FPR*) (Equation (13)) at various threshold values. The area under the curve (AUC) score quantifies the model’s overall ability to distinguish between positive and negative classes. A higher AUC score suggests superior model performance and generalization capabilities.

Equation (12). *TPR*.

(12) $$TPR=\frac{TP}{TP+FN}$$

Equation (13). *FPR*

(13) $$FPR=\frac{FP}{FP+TN}$$

### 3.2. Evaluation Results

Following the method described in Section 2.2, grid search cross-validation was used to optimize the RF model, finding that a configuration of 150 trees performed best. For this configuration, both the log^2^ and sqrt methods for determining the maximum number of features for node splitting achieved comparable accuracy. Additionally, the efficiency of the RF classifier was benchmarked against the eXtreme Gradient Boosting (XGBoost) algorithm using the same grid search cross-validation approach. Results indicated that RF outperformed XGBoost, with detailed hyperparameter optimization results for both methods provided in Supplementary Materials, File S1 (Spreadsheets 1 and 2). Following hyperparameter optimization, the RF model was selected as the best performing model, and the decision threshold (DT) was further tuned using the validation subset of the dataset. The optimal DT value was determined to be 0.53, as outlined in Supplementary Materials, File S1 (Spreadsheet 3). The final model was retrained by merging the training and validation subsets, after which it was employed to make predictions on the test subset of the large-scale PPI dataset.

The model’s predictive performance was evaluated using a confusion matrix, which provided insights into the proportion of accurate and inaccurate predictions per class. Standard evaluation metrics, including ACC, F1 score, PREC, and REC, as well as PREC/REC and ROC curves, were also calculated. The confusion matrix, along with detailed metrics and performance curves, is presented in Supplementary Materials, File S1 (Spreadsheet 4). In summary, the model demonstrated robust performance when applied to the large-scale PPI dataset, achieving 96.8% accuracy, 95.9% F1 score, 96.6% precision, and 95.2% recall. Notably, the area under the ROC curve (AUC) reached 99.2%, underscoring its strong discriminative power.

### 3.3. Experimental Verification

In addition, the model was evaluated on a curated subset of experimental host–gut bacterial protein interactions, where only 13 positive pairs have been verified so far, and thus no confirmed negative examples are available. The accuracy of the prediction is really high, which highlights the model’s ability to recapitulate biologically valid interactions (Table 2). Notably, the model’s posterior probabilities for these experimentally observed interactions span a range from approximately 0.39 up to 0.93, suggesting that the model’s confidence levels align reasonably well with known positive PPIs.

### 3.4. Network Visualization

**Figure 2:**
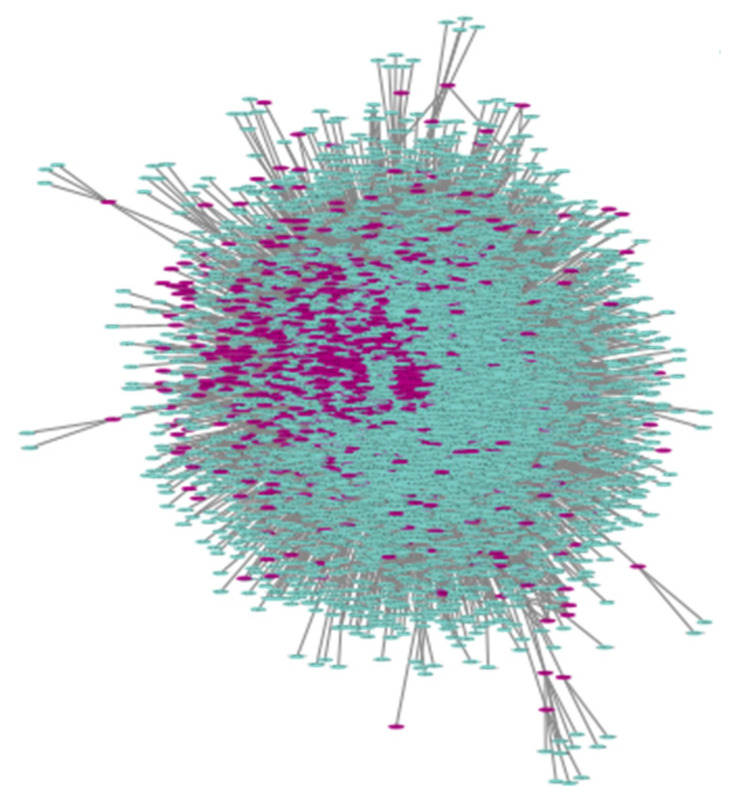
Visualization of the fuzzy human gut bacterial protein network. Purple nodes represent human protein clusters, and green nodes represent bacterial protein clusters.

The predicted network of the host–gut bacterial proteins consists of 432,511,851 interactions between 23,128 human and 399,579 bacterial proteins (422,707 nodes). The UniRef90 clustered network, which includes clusters of sequences that have at least 90% sequence identity and overlap with the longest sequence by at least 80%, contains 271,051,232 interactions between 16,669 human and 346,738 bacterial protein clusters (363,407 nodes). The final clustered network (fuzzy network), which was created by integrating proteins with 70% similar gene names and at least one identical Pfam ID, contains 456,555 interactions between 2177 human and 6863 bacterial protein clusters (9040 nodes) (Figure 2). The binary file (i.e., the file contains two columns: the bacterial and host protein clusters that interact) of the fuzzy network is contained in Supplementary Materials, File S2.

### 3.5. Network Centrality Analysis

The degree and integrated value of influence (IVI), a new metric of biological influence of each node within a network, of every cluster of the final network can be seen in Table 3 (human clusters) and Table 4 (bacterial clusters). The Gene Ontology (GO) terms of all the human fuzzy clusters are available in Supplementary Materials, File S3. From the bacterial proteins of the original network (pre-clustering network) that have the highest degree, the most influential bacterial strains of the GM interplay were also uncovered (Table 5). The results of all the networks’ analysis (cluster information, degree, IVI) can be found in Supplementary Materials, File S4.

### 3.6. Network Assortativity and Protein Disorder Analysis

**Figure 3:**
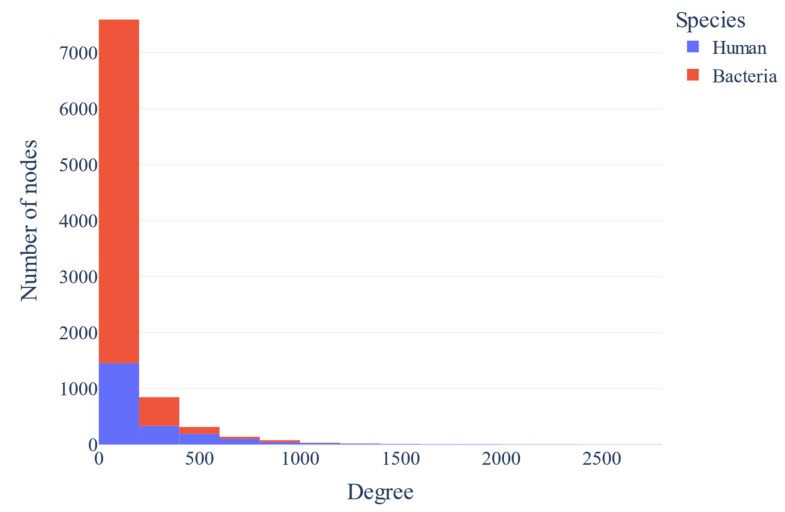
The degree distribution plot of nodes of the clustered network (blue: human proteins, red: bacterial proteins).

**Figure 4:**
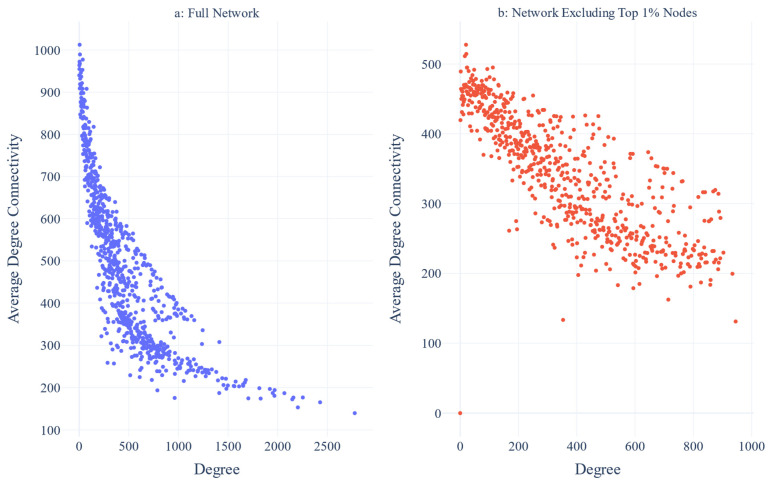
(**a**) The average degree of connectivity of nodes with degree k plot for the clustered network; (**b**) the average degree of connectivity of nodes with degree k plot for the clustered network, after excluding the top 1% most connected clusters.

**Figure 5:**
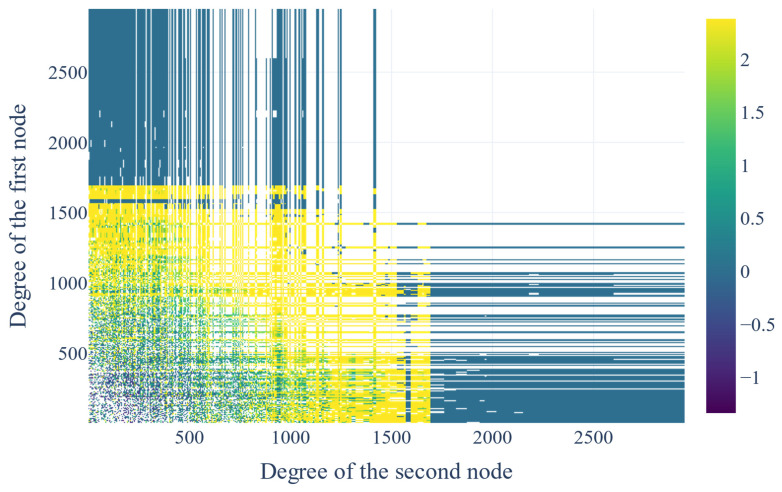
The correlation profile of the clustered network.

**Figure 6:**
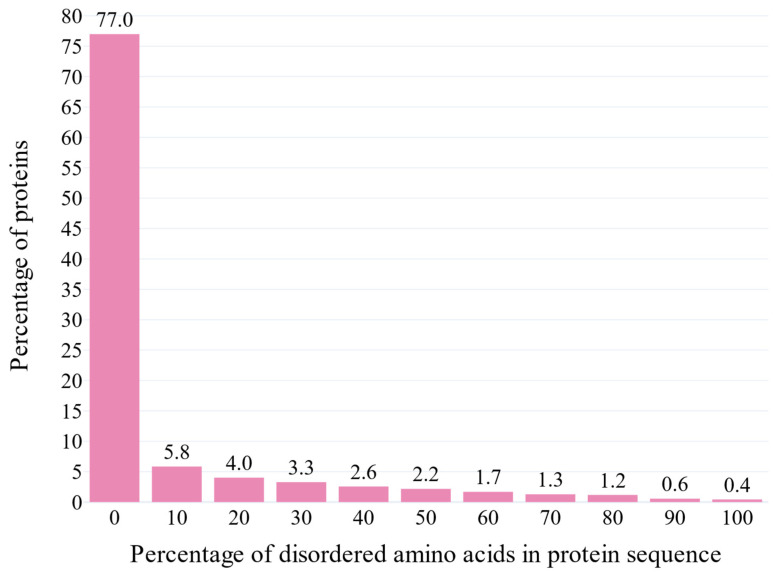
The percentage of proteins labeled as disordered in the dataset as a function of their percentage of disordered amino acids.

The assortativity coefficient of the clustered network is −0.34 and −0.29 after excluding the top 1% of the most connected clusters. The degree distribution plot is visualized below (Figure 3). The plot that correlated the average degree of connectivity of nodes with degree k (i.e., KNN(k), where nn: nearest neighbors) for the clustered network and the clustered network after excluding the top 1% most connected clusters are also depicted (Figure 4a,b). The correlation profile heatmap of the clustered network is visualized below (Figure 5). Additionally, the plots that visualize the intrinsic disorder of human proteins can be seen below (Figure 6).

### 3.7. Correlation of Protein Overall Centrality and Protein Centrality and Abundance

**Figure 7:**
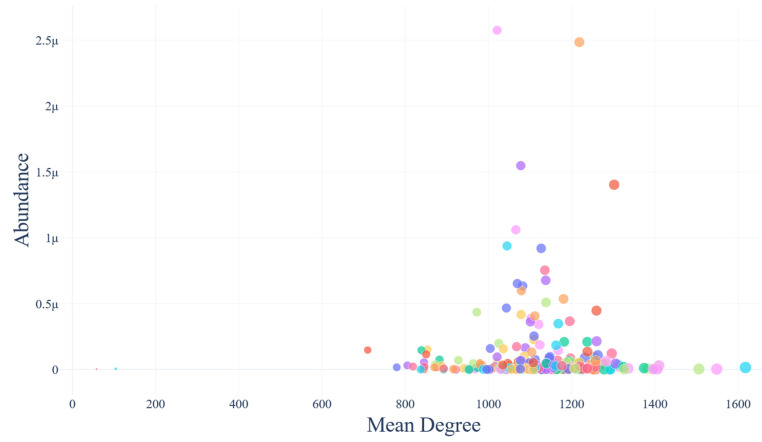
The mean degree–abundance association plot. Each bacterial strain is noted with a different color.

**Figure 8:**
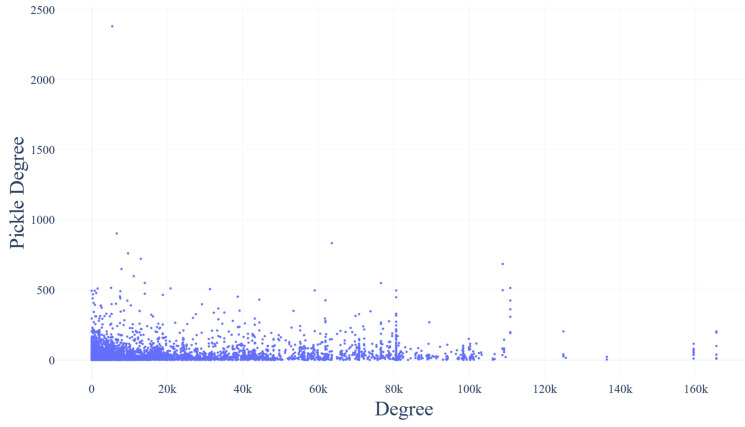
The protein degree in the human interactome (Pickle Degree)–protein degree in the human gut bacterial PPIN (Degree) association plot.

Below, two relationships are visualized: the association between each bacterial strain’s abundance and the mean degree of its proteins, and the correlation between the degree of each human protein in the host–gut bacterial network and its corresponding degree in the human interactome. (Figure 7 and Figure 8). The table with the abundance and mean degree per bacterial strain is available in Supplementary Materials, File S5. The Pearson correlation coefficient of the first plot is −0.003, whereas the one of the second plot is 0.1.

## 4. Discussion

### 4.1. Overview of the Predictive Model: Capabilities and Strengths

This study offers novel insights into the intricate interplay between the human host and gut microbiota via predicting their inter-species PPIN through statistical associations of Pfam domains. The ML model developed in this study showcases significant predictive capabilities for elucidating the gut microbiome–host protein interaction network. A core strength of the model is its capacity to handle the extensive datasets typical of protein interaction studies. A key component contributing to the model’s performance is the Pfam2Vec feature embedding, which leverages the Word2Vec methodology to transform Pfam domain identifiers into vector representations, providing a nuanced, context-aware encoding of domain information. This embedding enables the model to account for functional and structural similarities among proteins, enhancing predictive accuracy. Combined with the RF classifier, which is well regarded for its interpretability and resilience against overfitting, the model achieves both high precision and strong generalization capacity. This is evident from the high accuracy of the model in both the test subset of the large-scale experimental PPI dataset and the other test dataset that contained experimentally verified interactions between gut bacteria and human proteins located in the gut (i.e., case-study-specific test dataset). Notably, these strengths suggest that the framework could generalize to other biological contexts, which are not limited to cross-species protein interactions, offering an adaptable approach for further computational analyses in diverse biological domains.

### 4.2. Biological Significance of Predicted Interactions

#### 4.2.1. Bacterial Proteins with High Interaction Potential and Their Possible Influence on Host Pathways

From the network results, the bacterial proteins of the GM that are mostly interacting with the host proteins are serine/threonine kinases. New studies indicate that the gut microbiota could influence the function of kinase participating host pathways, like the mTOR [65] and CREB pathways [66], but also serve as biotargets for selective inhibitors to mediate microbiome-associated disorders [67].

##### The mTOR Pathway

Based on our results, in the predicted network, mTOR and the components of the mTOR complex 1 (mTORC1) and mTORC2 are central parts of the host–gut bacteria protein interplay. mTOR is a member of the class of serine/threonine kinases that detects and integrates a range of external and intracellular signals to preserve metabolic and cellular homeostasis [68]. Besides its function as a serine/threonine kinase, mTOR can also phosphorylate tyrosine residues [68]. mTOR is a part of two functionally distinct complexes, mTORC1 and mTORC2. On one hand, mTORC1 makes use of a multitude of growth factor and nutrient signals to promote cellular proliferation, particularly in situations where energy levels are sufficient and/or catabolism occurs in response to hunger [68]. mTORC1 also phosphorylates eIF4E Binding Protein (4EBP) and p70S6 Kinase 1 (S6K1), two important effectors that increase protein synthesis [69]. Furthermore, mTORC1 inhibition significantly affects mRNAs containing pyrimidine-rich 5′ TOP or “TOP-like” motifs, which are part of most genes involved in protein synthesis [70]. Additionally, mTORC1 stimulates de novo lipid synthesis through the sterol response element binding protein (SREBP) transcription factors that regulate the expression of metabolic genes involved in the manufacturing of fatty acids and cholesterol, but it also promotes a change in glucose metabolism from oxidative phosphorylation to glycolysis [69]. This protein complex additionally stimulates the synthesis of purine and pyrimidine nucleotides needed for DNA replication and ribosome biogenesis in developing and proliferating cells [69]. Finally, it inhibits protein catabolism via autophagy and thus it stimulates cellular development [69]. On the other hand, mTORC2 mainly regulates survival and proliferation by phosphorylating many protein kinases in the AGC (protein kinase A (PKA)/PKG/PKC) family, but the way it responds to environmental fluctuations is not yet elucidated [69]. mTORC2 is also a modulator of mitochondrial fitness, cytoskeletal modeling, and cell migration [71]. Compared to mTORC1, the role of mTORC2 in autophagy is less understood, but until now the evidence suggests that it is functionally different. More specifically, it acts as an autophagy suppressor when regulating mitochondrial permeability through the phosphorylation of serum and glucocorticoid-regulated kinase 1 (SGK-1) and when activating PKB (also known as AKT), which ultimately leads to mTORC1 activation [72]. The mTORC2/PKCα/β axis increases the rate of clathrin-dependent endocytosis, which in turn enhances autophagy [73]. Finally, mTORC2 signaling is implicated in the differentiation, metabolism, survival, activation, and function of a multitude of immune cells, including antigen-presenting cells (APCs), various T-cell subsets, and B-lymphocytes [72]. Since mTOR is so tightly connected with sensing nutrient and growth factors, it is no surprise that mTOR is activated in 60–80% of gastric cancer samples, and recently there has been genetic evidence that mTOR promotes proliferation and inhibits differentiation of gastric epithelial progenitors (GEPs) and gastric tumors [74,75]. Furthermore, mTOR has a prognostic value for lymph node metastasis, since its expression in metastatic lymph nodes is associated with poor clinical outcomes [76], but also a potential target for gastric cancer therapy [77]. It is also really interesting that the phosphoinositide 3-kinase (PI3K)/AKT/mTOR and mitogen-activated protein kinase (MAPK) pathways can also be activated by *Helicobacter pylori*, one of the major risk factors for gastric cancer that is responsible for merely 80% of cases [78].

##### The CREB Pathway

In the human gut protein network, CREB and many isoforms of adenylate cyclase participate in the predicted protein network. One of the most studied phosphorylation-dependent transcription factors is CREB, which is activated by a variety of serine-threonine kinases at serine 133. Following its phosphorylation, it binds to its coactivator protein, CREB-binding protein (CBP), or p300, to trigger the transcription of genes. Numerous biological processes, such as cell division, survival, proliferation, differentiation, adaptive responses, glucose homeostasis, spermatogenesis, circadian rhythms, and synaptic plasticity linked to memory and immune system function, have all been linked to CREB [79]. It has recently been proven that MAPK3/1–mTORC1 signaling is necessary for CREB activation and in turn promotes the expression of kit ligand (KITL) in pre-granulosa cells [80]. The pro-survival PI3K/Akt1/mTOR signaling pathway controls a variety of signal transductions and biological functions like transcription, protein synthesis, metabolism, autophagy, cell division, apoptosis, angiogenesis, and migration [80]. As part of this pathway, Akt functions by regulating transcription factors, like forkhead box O (FOXO), IκB kinase, and CREB, and suppresses pro-apoptotic while promoting anti-apoptotic BCL-2 factors as well as caspase inhibitors [80]. CREB1 is classified as a potential biomarker for tumor metastasis and patient outcome in gastric cancer [81]. In a subset of gastric cancers, carbonic anhydrase IX (Ca9) expression is maintained in the cancer cells at the invasion front, supporting the proposition that elevated Ca9 expression could play a role in promoting invasion, thereby contributing to the advancement of disease and tumor progression in these cancers [82]. Nevertheless, high Ca9 expression is a characteristic of healthy cells, and its loss is a frequent acquired feature of gastric cancer cells [82]. CREB has been shown to inhibit the transcription of CA9 in gastric cancer [83].

#### 4.2.2. Host Proteins Highly Targeted by the Microbiota

##### HSP Isoforms

The human proteins that are mostly targeted by the GM are mainly heat-shock proteins (HSPs), involved mainly in protein refolding and protein translocation [84]. Studies have shown that the enteric bacteria are one of the major regulators of HSP expression in the gut [85]. These chaperones not only protect the gut epithelium barrier against inflammation and oxidative stress, but are also responsible for refolding of misfolded proteins, degradation of unstable proteins, and transportation of proteins between different cellular components [86]. Many HSP isoforms, including HSP60, HSP70, and HSP90, are predicted to interact with a great number of gut bacterial proteins. Research on HSP expression throughout the GI tract highlights significant variation in localization and concentration, correlating with health status and regional challenges. Specifically, higher levels of HSP27 and HSP70 are noted in the stomach and large intestine, environments characterized by extreme acidity and diverse microbiota, respectively, unlike the proximal small intestine where these proteins are minimally expressed due to lower microbial diversity [85]. The expression pattern is influenced by dietary factors, microbes, and their metabolites, showcasing a clear dependency on the gut’s microbial composition, as evidenced by altered HSP levels in antibiotic-treated and germ-free mice [85]. However, high levels of HSP25 and HSP70 were documented in the colon of the non-antibiotic treated mice, suggesting a protective role against toxin A of *Clostridium difficile* [87]. Recent studies reveal intricate interactions between HSP and gut microbiota, notably the correlation of ileal HSP70 expression with *Lactobacillus* spp. abundance, emphasizing the role of probiotic strains in enhancing intestinal barrier integrity and epithelial cell protection through the induction of HSP27 and HSP70 [85]. Furthermore, a relationship between colonic HSP70 and *Clostridia* highlights the potential immunomodulatory effects of these bacteria, including the induction of anti-inflammatory cytokines like interleukin 10 (IL-10), suggesting HSP70’s involvement in immune regulation [85]. Additionally, the interaction between HSP gp96 (also known as HSP90B1) and microbiota is pivotal, especially for its role in toll-like receptor 4 (TLR4) activation, indicating a complex regulatory impact on immune responses [88]. High levels of HSP60, but not HSP90, have also been reported in IBD patients, as well as HSP autoantibodies [89]. It remains unclear why these antibodies form, with the most prevalent theories being that either they are in reality antibodies to the mycobacterial 65 kDa HSP (HSP60) that can also react with the host’s homologous protein, or they are a direct consequence of the native HSP release after gut epithelium injury [89]. Finally, HSP70 has displayed a protective role against atrophic and *Helicobacter pylori*-induced gastritis. In the case of cancer, however, HSP70 inhibition by quercetin or anti-sense HSP70 constructs works as an apoptotic sensitizer to colon cancer tumors [90,91].

##### Hypoxia-Regulated Proteins

There is compiling evidence that the host–microbiota interactions bidirectionally influence the hypoxia-regulated proteins, which sense and respond to changes in oxygen (O_2_) concentrations [92]. Interestingly, in this predicted protein network, hypoxia-inducible factor (HIF) 1-alpha and HIF-3a, which is not well characterized, are some of those hypoxia-regulated proteins that are part of the host–bacteria protein interactome. The digestive tract presents with a unique oxygen gradient, where the decrease in O_2_ concentration occurs in the direction of the small to large intestine and in the radial direction from the submucosal plexus into the intestinal lumen [93]. HIF is a necessary regulatory molecule of the intestinal homeostasis, since it up-regulates the tight junction proteins towards a more resistant epithelial barrier that promotes microbial colonization due to the reduction in gut inflammation [92]. This innate intestinal hypoxia is additionally stimulated by microbiota-derived metabolites like short-chain fatty acids, which further augment O_2_ consumption by intestinal epithelial cells (IECs) [84]. Two of the most studied HIFs are HIF-1α and HIF-2α, whose activities are complementary, with the first one mostly regulating the transcription of genes implicated in metabolic reprogramming, and the latter genes involved in angiogenic extracellular signaling, guidance cues, and extracellular matrix remodeling factors [94]. HIF-1α is implicated in multiple signaling pathways, such as the aforementioned PI3K/Akt/mTOR, which regulates the HIF-α mRNA levels [95]. In patients with inflammatory bowel syndromes, like Cron’s disease, the expression of both hypoxic factors HIF-1α and HIF-2α is also higher, resulting in a more hypoxic enteric mucosa [95]. According to recent studies, HIF-1 can be employed therapeutically to target signaling pathways linked to intestinal diseases with hypoxia [96]. Some specialists claim that HIF-2 has a greater pro-inflammatory function [96], and some inhibitors of HIF-2α, which could serve as therapeutic interventions in IBD and CRC, have been characterized [97]. Nevertheless, there is a need for better determination of the unique and shared functions of both HIFs for a better understanding of their contribution in normal intestinal function, developing pathologies, and therapeutics.

##### p53 Signaling Axis

Serine/threonine-protein kinase (Chk2), mouse double minute 2 homolog (Mdm2) (also known as E3 ubiquitin-protein ligase), and a variety of caspases that are part of the p53 signaling pathway also participate in the predicted human gut bacteria PPIN. It has been reported that the gut bacteria can influence the function of p53 in intestinal carcinogenesis [98]. Tumor protein 53 (TP53) contains domains related to transcriptional activation, DNA binding, and oligomerization, while displaying its tumor-suppressor function through regulating the decision making between the induction of programmed cell death, cell arrest, and repair or replicative senescence [99]. Both germline and somatic mutations of the tp53 gene contribute to the pathology of human cancer in different ways. In the first case, inherited mutations of the gene characterize Li-Fraumeni (LFS) and Li-Fraumeni-like (LFL) syndromes, and are the main cause of predisposition to early-onset cancers [100]. In the second case, somatic mutations of tp53 account for at least 50% of adult human malignancies [101] and many cases of colon cancers leading to poor prognosis [102]. The mutations of tp53 that lead to cancer formation can either be loss- or gain-of-function, with the first limiting the protein’s tumor-suppressing activity and the latter converting the protein to oncogenic [103]. Two of the most frequent mutations of the human tp53 gene are R172H and R270H, which display an enhanced tumor-suppressive behavior compared to the wild type. This tumor-suppressive function of the mutants was completely eradicated and turned into oncogenic after interacting with native gut microbiota [104]. Another study on zebrafish tp53^e7/e7^ mutant larvae demonstrated that this mutation contributes to disrupted sialic acid metabolism [105], which involves acids that display a variety of roles at the interface of host and microbiota interactions in the gut [106]. The gut of a zebrafish tp53^e7/e7^ mutant model presented with increased intestinal inflammation that was driven by microbiota dysbiosis, and more specifically the selective blooming and colonization of pro-inflammatory pathobionts such as *Aeromonas* spp., and further facilitated by an excess supply of intestinal Neu5Gc (i.e., a sialic acid), thus promoting pathobiont overgrowth [105]. Furthermore, *Akkermansia*, a mucin-degrading anaerobe that improves the intestinal mucosal barrier and possesses anti-inflammatory qualities, is significantly reduced in p53-knockout mice following radiation treatment, which indicates that p53 also has the ability to shape the GM [107].

##### DNA and RNA Helicases, Polymerases, and Proteasomal Components

DNA and RNA helicases, as well as DNA polymerases and subunits of the proteasome, are also central proteins of the predicted protein network. Recent studies have shown that DNA damage, which is an integral part of intestinal malignancies, can result both from reactive oxygen species (ROS) and reactive nitrogen species (RNS), but also from DNA damaging agents or toxins produced by the gut bacteria [108]. Genotoxins are chemicals or agents that damage DNA or chromosome structure released by the gut microbiota [109]. These toxins can induce double-strand breaks to the DNA of host epithelial cells and briefly stop the cell cycle [109]. Some of the most important genotoxins are Colibactin of *Escherichia coli* [110], cytolethal distending toxin (CDT) of *Campylobacter jejuni* [111], indolimines of *Morganella morganii* [112], and *Bacteroides fragilis* toxin *of Bacteroides fragilis* [113], which can result in mutations that directly lead to colorectal cancer. The bacteria of the GI tract can also cause mutations to the host’s DNA via the production of ROS, like *Enterococcus faecalis*, which produces extracellular superoxide, and *Helicobacter pylori*, which increases spermine oxidase in the host, both augmenting the chance of DNA mutation and resulting in carcinogenesis [109]. Moreover, the microbiota populations in the gut also influence the DNA integrity of the host, like in the case of pathogenic mutations in the APC tumor-suppressor gene, which were more frequent in patients with bigger populations of *Fusobacterium mortiferum* and less frequent in patients with smaller *Faecalibacterium prausnitzii* and *Bifidobacterium pseudocatenulatum* populations [114].

##### Viral Infection Pathway-Related Proteins

Finally, transcription factors, small and large ribosomal subunits, exportin-1, and other transport proteins whose functions are related to the viral infection pathway are targeted by the bacterial GM’s proteins. It has been previously demonstrated that the establishment and pathogenicity of viral infections are significantly influenced by the type and balance of surrounding commensal bacteria and vice versa [115]. Viral infections such as transmissible gastroenteritis virus (TGEV), rotavirus (RV), and Theiler’s murine encephalomyelitis virus lead to significant alterations in the intestinal microbiota across different animal models and clinical studies, characterized by a decrease in beneficial bacteria like *Lactobacillus*, *Bacteroidetes*, and *Firmicutes*, and an increase in potentially pathogenic families such as *Enterobacteriaceae*, *Escherichia*, and *Streptococcus*, which may contribute to secondary infections and higher mortality rates [116]. Furthermore, these fluctuations in microbial populations are found to vary with the disease phase, indicating a dynamic interplay between viral pathogenesis and microbiota composition [116]. In coronavirus disease 2019 (COVID-19) patients, opportunistic pathogens including *Actinomyces*, *Erysipelaclostridium*, *Streptococcus*, *Veillonella*, *Rothia*, and *Enterobacter* were associated with symptom severity, while *Faecalibacterium*, *Anaerostipes*, and *Bifidobacterium* were inversely correlated with disease severity [117]. In patients with respiratory syncytial virus (RSV) or influenza virus infection, inappetence is a common symptom that results in changes in the GM and metabolome, with a significant increase in lipids, which is ultimately related with resolution of the disease [118]. On one hand, the segmented filamentous bacteria of the gut can also strengthen the host’s resistance to viruses like RV by enhancing the multiplication, migration, and shedding of the epithelial cells of the intestines [119]. On the other hand, the intestinal bacteria can promote viral infection via stabilizing the virion structure, mediating the recombination of viruses, augmenting the differentiation of the target cells, and weakening the host’s immune response [116]. Supplementary Materials, File S6 contains the host’s proteins that target the GM’s proteins and are also targeted by it, which are part of the predicted protein interaction network.

### 4.3. Network Topology and Properties of the Host–Gut Bacteria PPIN

#### 4.3.1. Degree Distribution and Scale-Freeness

From the degree distribution plot (Figure 3), it can be deducted that the P(k) of predicted clustered protein network displays a scale-free behavior, since it follows a power law, i.e., P(k) ~ k^−γ^. In this case, it seems that 2 < γ < 3, which is the typical value of γ [120]. This distribution indicates that while most proteins in the network have a limited number of interactions, and a few “highly connected” proteins form essential hubs that interact with many other proteins. Preferential attachment (i.e., new nodes prefer to link to nodes with higher degrees) and growth (i.e., the number of nodes in the network increases over time) contribute to the emergence of the scale-free property in complex networks [120]. It is hypothesized that the scale-free character of the degree distribution is at least partially due to gene duplication events, because it is thought to underlie the phenomenon of preferential attachment [121].

#### 4.3.2. Assortativity and Degree Correlation

Based on the assortativity coefficient, the network appears to be disassortative, as the value of this Pearson coefficient is negative. However, when the top 1% most connected clusters are removed, and the coefficient is measured again, it becomes more positive, which is not expected from a true disassortative network where the value should become more negative. Due to this, more metrics measuring the assortativity of the network were evaluated, to get a better picture of this network’s organization. From the KNN(k) plot, we can see that KNN follows a decreasing function of k, a behavior that is indicative of disassortative networks (Figure 4a). However, after excluding the top 1% most connected clusters, the function becomes less decreasing, which suggests an assortative correlation (Figure 4b). Finally, the diagonal in the correlation profile (Figure 5) provides direct proof of dichotomy in degree correlation. In assortative and disassortative networks, the diagonal of the plot is similar in color and is indicative of the degree correlation pattern of the network, where nodes with similar degrees either attract (yellow) or repulse (blue) each other, respectively. On one hand, a disassortative network is distributed by hub repulsion, which suggests a modularity model with nodes structured around scattered hubs, in turn producing better-linked but susceptible networks. On the other hand, an assortative network is made up of fully connected hubs, which leads to less connected but resistant networks [60]. Since biological networks are neither disassortative nor assortative, the protein networks in living cells have the advantages of both types of networks while, at the same time, they avoid the drawbacks [60].

### 4.4. Intrinsic Disorder Analysis

#### 4.4.1. Disordered Regions in Host vs. Bacterial Proteins

From the intrinsic disorder plots (Figure 6), it is evident that 25% of all the human proteins participating in the network have some disordered amino acids, while the corresponding percentage of their bacterial counterparts is less than 2%. Apart from their roles in signal transduction [122], disordered proteins also participate in a variety of key biological tasks, including being post-translational modification sites, mediating the phase separation process [123], nucleic acid binding [124], and the entropic chain mechanism, which is responsible for fast shifts among several different protein conformations [125]. There is a considerable positive association between the proportion of disordered regions and organism complexity [126]. Consequently, intrinsic disorder is more abundant in eukaryotic proteins [127], in the form of short linear motifs (SLiMs), which have a length of 3–15 amino acids and provide flexibility and accessibility for interactions [128].

#### 4.4.2. Functional Implications of Intrinsically Disordered Proteins

Most of the time, these disordered regions are also characterized by their low complexity and charged residue content, making them common in membrane signaling receptors [127]. Besides their roles in normal function, disordered proteins are also involved in numerous serious disorders in humans, such as neurodegenerative (Aβ protein, Tau Protein, prion proteins, a-synuclein) [129], cancer (breast cancer type 1 susceptibility protein (BRCA-1) and p53), cardiovascular disease (thrombin), and diabetes (amylin) [130,131]. After protein comparison between eukaryotic and prokaryotic proteomes, it was proven that the linker sections between Pfam domains are the source of the intrinsic disorder difference [130]. More specifically, in the case of eukaryotic proteins, the linker regions are more extensive, and they also have a bigger percentage of disordered amino acids [130]. At the same time, eukaryotic proteins contain a higher percentage of serine and proline, and a lower percentage of isoleucine, than procaryotic ones, but it is not yet clear if these changes are a cause or a direct consequence of the increased disorder [130]. It should be noted that most of the proteins in the bacterial strains are part of the unreviewed (TrEMBL) registrations of the UniProt database, which are high-quality computationally evaluated records enhanced with automatic annotation and classification. Their lack of extensive annotation could justify the fact that there is not much available information about their intrinsic disorder. Nevertheless, based on the literature, intrinsically disordered proteins may only account for 2–5% of typical bacterial proteomes, but some of those proteins are essential for a multitude of functions of bacterial biology [132]. Recent research indicates that pathogenic bacteria release effector proteins and have intrinsic disordered regions, like the cytotoxin-associated gene A (CagA) effector of *Helicobacter pylori* and the adenylate cyclase (CyaA) effector of *Bordetella pertussis*, which interacts via the host calmodulin and works as an oncogenic scaffold protein interacting with a wide range of host signaling molecules associated with carcinogenesis [133]. A recently characterized effector of the latter bacterial strains is the translocated intimin receptor (Tir), which possesses host-like disordered protein regions that serve as a fruitful strategy to subvert host eukaryotic systems and promote infection [127]. Other important effector proteins include the effectors *E. coli* secreted protein (Esp) F(U))/tir-cytoskeleton coupling protein (TccP) and EspB of *Enteropathogenic* and *Enterohemorrhagic Escherichia coli* strains, respectively [127]. EspF targets and degrades the host’s mitochondria by consuming DNA repair proteins and TccP couples Tir to the actin-cytoskeleton [109,134]. Finally, EspB blocks the interaction of myosins with actin and leads to myosin inhibition to facilitate the infection by the pathogen [135].

### 4.5. Key Bacterial Strains and Their Clinical Relevance

#### 4.5.1. Most Influential Bacterial Strains

Based on the most influential bacterial strains (Table 2), *Peptoniphilus harei* is an anaerobic Gram-positive bacterium that is part of the gut and vaginal microbiota, but it is also involved in polymicrobial infections in pressure ulcers, osteoarticular samples and skin, and soft-tissue infections [136,137]. There is also a case of peritoneal infection in a patient with an occlusive syndrome and another case of bacteremia in an abdominal aortic aneurysm patient caused by *Peptoniphilus harei* [136,138]. Additionally, *Peptoniphilus harei* has been documented to be tetracycline resistant [139]. *Peptostreptococcus anaerobius* is augmented in the gut of colorectal cancer patients compared to healthy individuals [140,141], but it also involved in polymicrobial and monobacterial infections, such as the abdominal cavity, soft tissue, brain, bone, and respiratory and urogenital tracts [142]. *Coprococcus eutactus* has been reported to be depleted in patients with Parkinson’s disease and children with delayed language development in Uganda [143,144]. Furthermore, *Coprococcus eutactus* serves as a potent probiotic for the amelioration of colitis symptoms [145]. *Aeromonas veronii*, according to a study in Taiwan, is the predominant bacteria in the stools of patients [146], but also a pathogen causing gastroenteritis and one of the bacteria detected in IBD [147]. Furthermore, *Aeromonas veronii* has been documented in cases of bacteremia [148]. There have also been several cases of immunocompromised patients with necrotizing fasciitis [149], community-acquired pneumonia [150], and soft tissue infection [151], and a case of infection following a biliary drainage [152]. It is evident that the most prominent bacteria of the gut not only influence the gut system and gut-related pathologies, but also many other systems within the human body, indicating the holistic influence of the gut microbiota in human health.

#### 4.5.2. Strain Abundance vs. Network Connectivity

Since the Pearson correlation coefficient of the plot that associates the abundance percentage with the mean degree of every bacterial strain (Figure 7) lies around −0.003, there appears to be no correlation between the abundance and the number of first neighbors (i.e., the degree) of the gut bacterial proteins. This is a very important finding, as it suggests that some of the most influential proteins of the network could very likely belong in strains that are not part of the most abundant ones, indicating that these strains’ proteins can also be key players of the human bacterial gut PPIN. Additionally, it is evident that for the establishment of the host’s health, gut biodiversity is crucial. Finally, based on the plot that associates the degree of the human proteins that participate in the host–gut interplay versus in the human interactome (Figure 8), there appears to be a non-significant correlation between the two. The latter highlights that the most influential regulators of this inter-species network are not the same as the ones that highly influence the host’s protein interactions, suggesting a functional independence of this system.

## 5. Conclusions

This study has contributed to the better understanding of the intricate interplay between the human host and gut bacterial microbiota through the prediction of inter-species PPIs based on statistical associations of Pfam domains. Our comprehensive network analysis revealed over 432 million interactions between human and bacterial proteins, shedding light on the vast complexity of the GM–host relationship and highlighting key regulatory molecules and pathways influenced by this dynamic system. As the host’s pathways that include the human proteins found in the predicted PPIN have been previously found to be influenced by the gut bacteria, our results can serve as a possible molecular explanation of the host–gut bacteria interaction at the protein level. Moreover, our results emphasize the crucial role of gut biodiversity in the maintenance of the host’s overall health, revealing that the most impactful proteins do not always belong to the most abundant bacterial strains. The analysis of the intrinsic protein disorder showed that the host’s proteins have a far greater percentage of disordered regions, probably due to their complex biological roles. Finally, the results demonstrated the functional independence of the gut–host PPIN from the human interactome, suggesting a unique regulatory mechanism underlying the bacterial GM–host interplay.

The presented model of the complex interplay between the proteins of the gut bacteria and the host lays the groundwork for future experimental validation. By employing advanced proteomic techniques such as mass spectrometry-based cross-linking assays and yeast-two-hybrid systems, the most probable statistical predictions can be verified. Additionally, comparative analyses of interaction patterns across various health and disease states could elucidate the molecular underpinnings of gut-related pathologies. These experimental endeavors will not only validate computational models but also provide actionable insights for the development of targeted therapeutic interventions aimed at modulating specific gut–host interactions for improved health outcomes. By providing a predictive framework and identifying key regulatory elements, this study opens new avenues for understanding the GM’s holistic role in health and disease by contributing to the design of targeted and more efficient experiments that are of utter importance for the development of specific interventions that can modulate these interactions for personalized therapeutic benefits.

## Figures and Tables

**Table 1:** Dataset distribution.

Subset	Positive	Negative	Total
Training	728,416	621,010	1,349,426
Validation	84,395	128,929	213,324
Test	118,076	180,972	299,048

**Table 2:** The probability of interaction of experimentally verified host–gut bacteria protein interactions. Each protein is symbolized with its corresponding UniProt ID.

P1	P2	Probability
P0A6B7	P16070	0.68
P0AA25	P24941	0.65
P04949	P51617	0.45
P00634	Q5S007	0.47
P04949	Q13233	0.45
P0AA10	Q9NX20	0.84
P0AA10	Q9BYD3	0.93
P04949	Q99836	0.39
P0AD57	Q86YH6	0.82
P0A6F5	Q16762	0.69
P16525	P68366	0.87
P0A6H1	Q16740	0.80

**Table 3:** The ten most influential human protein clusters of the fuzzy network.

Human Cluster	IVI	Degree	Protein Description
fc_A0A7I2V2S7	100	2776	Stress-70 protein, mitochondrial
fc_E7ERF2	97	2203	T-complex protein 1 subunit alpha
fc_P63104	83	1828	14-3-3 protein zeta/delta
fc_P62917	72	1704	Large ribosomal subunit protein uL2
fc_A0A6I8PR89	68	2158	DnaJ heat shock protein family (Hsp40) member C7
fc_Q15029	66	2147	116 kDa U5 small nuclear ribonucleoprotein component
fc_A0A2R8Y425	63	2427	DNA helicase
fc_O60229	62	1967	Kalirin
fc_Q08211	56	1950	ATP-dependent RNA helicase A
fc_P62701	56	1483	Small ribosomal subunit protein eS4, X isoform

**Table 4:** The ten most influential bacterial protein clusters of the fuzzy network.

Bacterial Cluster	IVI	Degree	Protein Description
fc_A0A225U972	32	754	ATP-binding protein
fc_A9KS63	29	898	Extracellular solute-binding protein family 1
fc_A0A6A7LXF3	29	915	Protein kinase
fc_A0A833CCQ3	29	917	DUF805 domain-containing protein
fc_R7M519	29	917	non-specific serine/threonine protein kinase
fc_R5GGG2	29	917	non-specific serine/threonine protein kinase
fc_H1DES1	29	917	Protein kinase containing protein
fc_R5LKV6	29	923	HRDC domain protein
fc_G1V1Z8	29	878	RRM domain-containing protein
fc_A0A0P0G9P4	29	878	RRM domain-containing protein

**Table 5:** The ten most influential bacterial strains.

UniProtID	Organism Name
A0A2X1YLR3	*Peptoniphilus harei*
A0A1Y4LZH0	*Faecalitalea cylindroides*
A0A921MAI4	*Phocaeicola barnesiae*
K1J3I2	*Aeromonas veronii* AMC34
A0A135YM99	*Peptostreptococcus anaerobius*
R7LZ16	*Fusobacterium* sp. CAG:815
R7LW02	*Acidaminococcus* sp. CAG:542
R7MMQ3	*Ruminococcus* sp. CAG:624
A0A173WGW7	*Coprococcus eutactus*
R6RRB6	*Firmicutes bacterium* CAG:449

## Data Availability

The original contributions presented in this study are included in the article/Supplementary Material. Further inquiries can be directed to the corresponding author(s).

## Supplementary Materials

The following supporting information can be downloaded at: https://www.mdpi.com/article/10.3390/biotech14010013/s1, Spreadsheets S1: Evaluation Results; Tab-seperated File S2: Fuzzy Cluster Cluster Interactions; Spreadsheet S3: GO Term Analysis; Spreadsheets S4: Aggregated Results for the predicted networks; Comma-seperated spreadsheet S5: Bacteria Information for Healthy and Unspecified diseases; Spreadsheet S6: Proteins in Network Pathways.

## Abbreviations

The following abbreviations are used in this manuscript:

GM	Gut microbiome
GI	Gastrointestinal
IBDs	Intestinal bowel diseases
IBS	Irritable bowel syndrome
NPD	Neuropsychiatric disorders
ASD	Autism spectrum disorder
ADHD	Attention-deficit/hyperactivity disorder
SCZ	Schizophrenia
RA	Rheumatoid arthritis
SpA	Spondyloarthritis
SLE	Systemic lupus erythematosus
MS	Multiple sclerosis
CRC	Colorectal cancer
MS	Mass spectrometry
ML	Machine Learning
PPIs	Protein–protein interactions
PPIN	Protein–protein interaction network
mTOR	Mammalian target of rapamycin
CREB	CAMP response element-binding protein
IVI	Integrated Value of influence
GO	Gene Ontology
mTORC1	MTOR complex 1
4EBP	EIF4E Binding Protein
S6K1	P70S6 Kinase 1
SREBP	Sterol response element binding protein
SGK-1	Serum and glucocorticoid-regulated kinase 1
APC	Antigen-presenting cells
GEPs	Gastric epithelial progenitors
PI3K	Phosphoinositide 3-kinase
MAPK	Mitogen-activated protein kinase
CBP	CREB-binding protein
KITL	Kit ligand
FOXO	Forkhead box O
Ca9	Carbonic anhydrase IX
HSPs	Heat-shock proteins
IL-10	Interleukin 10
TLR4	Toll-like receptor 4
O2	Oxygen
HIF	Hypoxia-inducible factor
IECs	Intestinal epithelial cells
Chk2	Serine/threonine-protein kinase
Mdm2	Mouse double minute 2 homolog
TP53	Tumor protein 53
LFS	Li-Fraumeni syndrome
LFL	Li-Fraumeni-like
*E. coli*	*Escherichia coli*
ROS	Reactive Oxygen Species
RNS	Reactive Nitrogen Species
CDT	Cytolethal distending toxin
TGEV	Transmissible gastroenteritis virus
COVID-19	Coronavirus disease 2019
RSV	Respiratory syncytial virus
RV	Rotavirus
SLiMs	Short linear motifs
BRCA-1	Breast cancer type 1 susceptibility protein
CagA	Cytotoxin-associated gene A
CyaA	Adenylate cyclase
Tir	Translocated intimin receptor
Esp	E. coli secreted protein
TccP	Tir-cytoskeleton coupling protein
API	Application Programming Interface
DDIs	Domain–domain Interactions
EBI	European Bioinformatics Institute
RF	Random forest
TP	True positives
TN	True negatives
FP	False positives
FN	False negatives
ACC	Accuracy
PREC	Precision
REC	Recall
ROC	Receiver operating characteristic
TPR	True positive rate
FPR	False positive rate
AUC	Area under the curve
DT	Decision threshold
MMseqs2	Many-against-many sequence searching
PICKLE	Protein interaction knowledgebase
